# More Than Just Static: Dynamic Functional Connectivity Changes of the Thalamic Nuclei to Cortex in Parkinson's Disease With Freezing of Gait

**DOI:** 10.3389/fneur.2021.735999

**Published:** 2021-10-15

**Authors:** Shangpei Wang, Huanhuan Cai, Zong Cao, Chuan Li, Tong Wu, Fangcheng Xu, Yinfeng Qian, Xianwen Chen, Yongqiang Yu

**Affiliations:** ^1^Department of Radiology, The First Affiliated Hospital of Anhui Medical University, Hefei, China; ^2^Research Center of Clinical Medical Imaging, Hefei, China; ^3^Anhui Provincial Institute of Translational Medicine, Hefei, China; ^4^Department of Neurology, The First Affiliated Hospital of Anhui Medical University, Hefei, China

**Keywords:** Parkinson's disease, freezing of gait, resting-state fMRI, thalamus, functional connectivity

## Abstract

**Background:** The thalamus is not only a key relay node of the thalamocortical circuit but also a hub in the regulation of gait. Previous studies of resting-state functional magnetic resonance imaging (fMRI) have shown static functional connectivity (FC) between the thalamus and the cortex are disrupted in Parkinson's disease (PD) patients with freezing of gait (FOG). However, temporal dynamic FC between the thalamus and the cortex has not yet been characterized in these patients.

**Methods:** Fifty PD patients, including 25 PD patients with FOG (PD-FOG) and 25 PD patients without FOG (PD-NFOG), and 25 healthy controls (HC) underwent resting-state fMRI. Seed-voxel-wise static and dynamic FC were calculated between each thalamic nuclei and other voxels across the brain using the 14 thalamic nuclei in both hemispheres as regions of interest. Associations between altered thalamic FC based on significant inter-group differences and severity of FOG symptoms were also examined in PD-FOG.

**Results:** Both PD-FOG and PD-NFOG showed lower static FC between the right lateral posterior thalamic nuclei and right inferior parietal lobule (IPL) compared with HC. Altered FC dynamics between the thalamic nuclei and several cortical areas were identified in PD-FOG, as shown by temporal dynamic FC analyses. Specifically, relative to PD-NFOG or HC, PD-FOG showed greater fluctuations in FC between the left intralaminar (IL) nuclei and right IPL and between the left medial geniculate and left postcentral gyrus. Furthermore, the dynamics of FC between the left pulvinar anterior nuclei and left inferior frontal gyrus were upregulated in both PD-FOG and PD-NFOG. The dynamics of FC between the right ventral lateral nuclei and left paracentral lobule were elevated in PD-NFOG but were maintained in PD-FOG and HC. The quantitative variability of FC between the left IL nuclei and right IPL was positively correlated with the clinical scales scores in PD-FOG.

**Conclusions:** Dynamic FC between the thalamic nuclei and relevant associative cortical areas involved in sensorimotor integration or cognitive function was disrupted in PD-FOG, which was reflected by greater temporal fluctuations. Abnormal dynamic FC between the left IL nuclei of the thalamus and right IPL was related to the severity of FOG.

## Introduction

Freezing of gait (FOG) is one of the most common motor symptoms experienced by patients with Parkinson's disease (PD) and is defined as a brief and sudden inability to initiate walking, despite the intention to step (1). FOG reduces mobility and significantly increases the risk of falls, which can have devastating consequences. Currently, the neural mechanisms underlying FOG are poorly understood, and no effective treatments are available. FOG is thought to arise from the impaired communication between a wide range of neural structures, which is reflected by the breakdown of an underlying neural network rather than by discrete lesions (2). Therefore, it is most appropriate to investigate the network circuity to understand the pathophysiology of FOG.

The thalamus is a comprehensive relay node for transmitting diverse information, which includes multimodal sensory inputs as well as projections from motor, cognitive, and limbic structures (3, 4). Moreover, the thalamus has reciprocal connections with extensive cortical and subcortical regions and functions as a subcortical processing hub for gating of multi-information and integrating of sensorimotor and cognitive functions. Thus, the thalamus is a central relay station and an integration center of the central nervous system. The thalamus, as a key node in the basal ganglia-thalamocortical and cerebello-thalamocortical circuits, plays a crucial role in the modulation of gait. Thalamic morphology predicts the onset of FOG in PD (5). Although isolated thalamus lesions seldom induce FOG, disruption of thalamic neural activity and functional connections contribute to the development of FOG. It is well established that dynamic motor circuit activity is altered in PD patients, which is reflected in the motor symptoms. A well-defined metabolic spatial covariance pattern, the PD-related pattern (6), revealed using principal component network analysis of data acquired from positron emission tomography (PET) imaging, showed increased activity in the pallidothalamic and pontocerebellar regions, which was associated with reduced activity in the premotor cortex and parietal association regions (7). As a key node of this neural network, the thalamus has been consistently shown to have increased metabolism across different PD populations and in primate PD models. Symptoms of PD are associated with subtle thalamic abnormalities, and neurosurgery of the thalamus provides symptom relief (8). In a PD rat model, deep brain stimulation of the ventral anterior and lateral thalamus was shown to improve the motor symptoms of bradykinesia, postural instability, and gait dysfunction (9). Furthermore, acetylcholinesterase PET imaging studies demonstrated that cholinergic thalamic changes are significant contributors to the freezing features of PD. FOG status was associated with reduced acetylcholine transporter expression in the right visual thalamus complex, which included the lateral geniculate nuclei (10).

Structural and functional neuroimaging studies have sought to characterize the thalamic impairment patterns of FOG. PD patients with FOG (PD-FOG) have been shown to have reduced thalamic volume (11). A functional study using PET to measure the cerebral metabolism of PD-FOG during a complex walking task known to elicit FOG episodes demonstrated that individuals with FOG have increased activation of the thalamus and motor areas and decreased activation of parietal areas (12). A functional magnetic resonance imaging (fMRI) study (13) in PD-FOG that employed a virtual reality gait paradigm indicated that the freezing phenomenon was associated with decreased blood oxygen level-dependent (BOLD) signal bilaterally in the head of the caudate nucleus, the thalamus, and the globus pallidus internus, which suggested an increase in basal ganglia inhibitory output and a decrease in thalamic and brainstem information processing. In contrast to task-based fMRI analyses, resting-state fMRI functional connectivity (FC), which requires no experimental task, can be used to study multiple neural systems simultaneously. Aberrant static FC is frequently reported in PD-FOG (14–17). Despite extensive evidence for abnormal regional and network activity in PD patients, studies focusing on the alterations of thalamocortical connectivity are scarce, especially in those with FOG. In addition, most studies have explored the thalamus as a single unit without considering the diverse structure and function of the various thalamic subdivisions. Given the heterogeneity in the cytoarchitecture, fiber connections, and functions of the thalamic subregions, alterations in FC of the individual thalamic nuclei with other brain regions may be distinctly affected in PD-FOG. To date, such investigations have not been conducted.

Because of the nature of constantly changing neural activity, increasing evidences have demonstrated that FC across brain regions is inherently dynamic, rather than simply static (18, 19). Static and dynamic FC detect different aspects of abnormal neural activity in PD and can offer complementary evidence (20, 21). To the best of our knowledge, there has not been any study investigating the dynamic FC of the thalamus and cortex, especially in the context of FOG symptoms in PD. The purpose of this study was to quantify the changes in static and dynamic FC of the individual thalamic nuclei with cerebrocortex in PD-FOG. We hypothesized that PD-FOG would exhibit altered static and dynamic thalamocortical FC with distinct thalamic nuclei-cortical FC patterns in contrast to PD-NFOG.

## Materials and Methods

### Participants

Fifty PD patients, including 25 PD-FOG and 25 PD-NFOG, were recruited from the inpatient and outpatient departments of The First Affiliated Hospital of Anhui Medical University. PD-FOG and PD-NFOG were matched for age, sex, duration of illness, and mini-mental status examination (MMSE) score. A cohort of 25 age- and sex-matched healthy controls (HC) without neuropsychiatric comorbidities or any family history of Parkinsonism were recruited. All subjects were right-handed. Exclusion criteria included magnetic resonance imaging (MRI) contraindications, presence of focal brain lesions on MRI, atypical Parkinsonian disorders, previous history of other neurological condition, psychiatric disorder, or musculoskeletal impairments that interfered with gait or balance, major medical illness within the previous 3 months, and inability to follow instructions. The study was approved by the ethics committee of The First Affiliated Hospital of Anhui Medical University, and written informed consent was obtained from all subjects.

### Clinical Assessment

PD patients were clinically diagnosed by a movement disorders specialist, according to the clinical diagnostic criteria of the United Kingdom PD Society Brain Bank (22). Parkinsonian motor symptoms were assessed using the Hoehn-Yahr staging scale and the Unified Parkinson's Disease Rating Scale part III (UPDRS III). Participants were included in the PD-FOG group only if FOG episodes occurred naturally or were induced by provocative test of rapid 360° turns (23) clockwise as well as counterclockwise in the consulting room. The new freezing of gait questionnaire (NFOGQ) (24) was used to quantitatively assess the severity of FOG. Anti-Parkinsonian dosages are reported as levodopa equivalents, which were calculated based on clinically equivalent dosing estimates (25). For each PD patient, the levodopa equivalent daily dose (LEDD) was estimated based on the anti-Parkinsonian drugs and dosages used in the week before the MRI scan. All tests were conducted after withholding anti-Parkinsonian drugs for more than 12 h during the practical “Off” levodopa state.

### Image Acquisition

MRI scans were acquired on a 3.0 T system (Discovery 750 w, GE, Milwaukee, WI) using a 24-channel head coil. Earplugs and tight and comfortable foam padding were used to reduce scanner noise and head motion. All participants were instructed to keep their eyes closed, relax, not fall asleep, think of nothing in particular, and move as little as possible during the scan. Three-dimensional high-resolution T1-weighted structural images were acquired using a brain volume (BRAVO) sequence. The parameters used were as follows: repetition time (TR) = 8.5 ms; echo time (TE) = 3.2 ms; inversion time = 450 ms; flip angle (FA) = 12°; matrix size = 256 × 256; field of view (FOV) = 256 × 256 mm^2^; sagittal slice number = 188; slice thickness = 1 mm, no gap; and total time of acquisition = 4 min and 56 s. BOLD resting-state fMRI data were acquired using a gradient-echo single-shot echo-planar imaging sequence. The parameters used were as follows: TR = 2,000 ms; TE = 30 ms; FA = 90°; matrix size = 64 × 64; FOV = 220 × 220 mm^2^; interleaved axial slice number = 35; slice thickness = 3 mm; slice gap = 1 mm; volume number = 185; and total time of acquisition = 6 min and 10 s.

### Image Data Preprocessing

All images were first visually inspected for visible artifacts. Resting-state fMRI data were preprocessed using Statistical Parametric Mapping 12 (SPM12, http://www.fil.ion.ucl.ac.uk/spm) and Data Processing and Analysis for Brain Imaging (DPABI, http://rfmri.org/dpabi) (26). To allow the signal to reach equilibrium and participants to adapt to the scanning noise, the first 10 volumes were removed. The remaining 175 volumes were corrected for the acquisition time delay between slices. To correct for head motion between the different time points, realignment was performed. To quantitatively index head motion, frame-wise displacement (FD) between volumes was calculated. The motion thresholds for the translational direction and axis angular rotation were defined as 3 mm and 3°, respectively. The estimated motion parameters based on the Friston-24 model (i.e., linear drift, cerebrospinal fluid signal, and white matter signal) were regressed out of the data as nuisance covariates. The datasets were then filtered with a frequency range of 0.01 to 0.1 Hz. A high-level non-linear warping algorithm called the diffeomorphic anatomical registration through the exponentiated lie algebra (DARTEL) technique (27) was used to perform normalization. Specifically, individual structural images were first co-registered to the mean functional image, and the transformed structural images were then segmented and normalized to the Montreal Neurological Institute (MNI) space. Using the estimated deformation parameters, the voxel size of each filtered functional volume was spatially normalized to MNI space and resampled to 3 mm. Before statistical analysis, Gaussian smoothing was applied to the data at 6 mm full width at half maximum.

### Definition of the Thalamic Seeds

**Figure 1:**
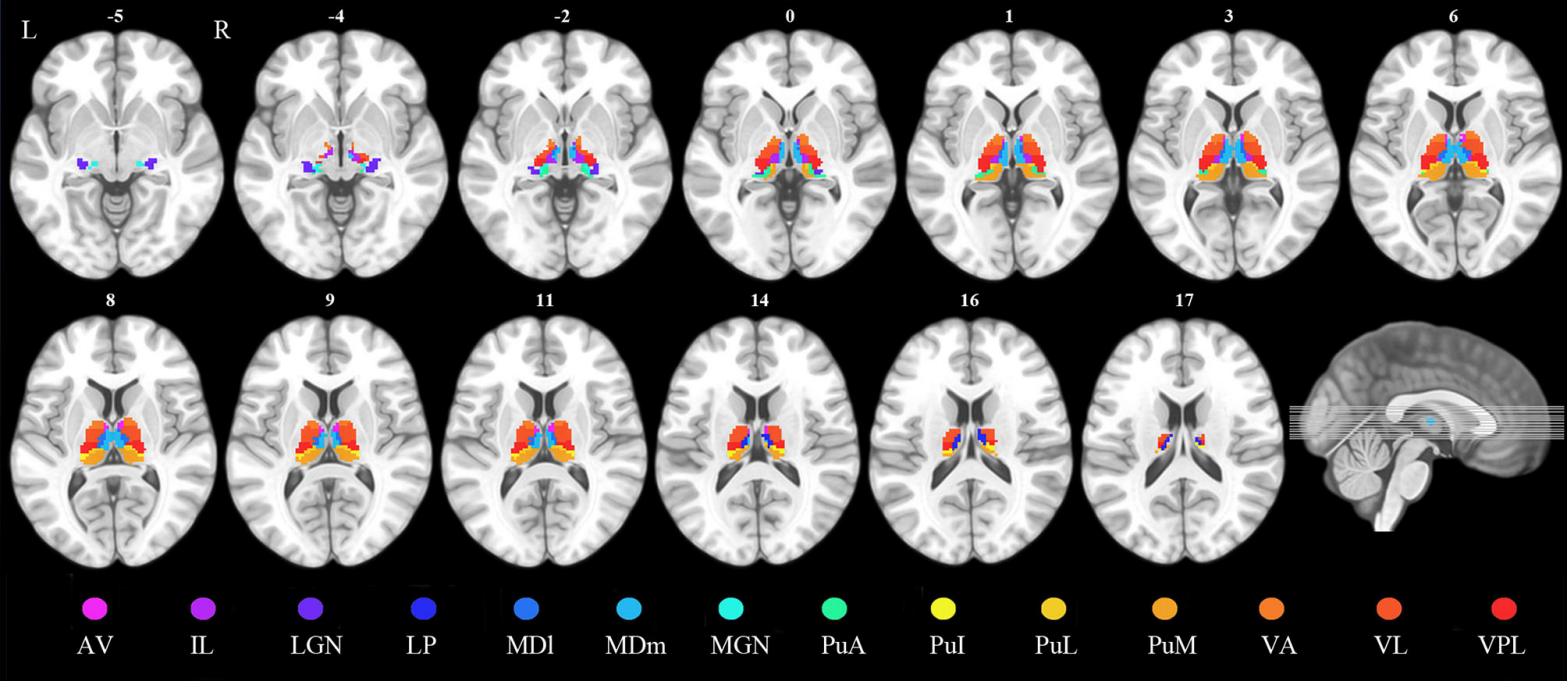
Definition of the thalamic nuclei. AV, anteroventral nuclei; IL, intralaminar nuclei; LGN, lateral geniculate; LP, lateral posterior nuclei; MDl, mediodorsal lateral parvocellular nuclei; MDm, mediodorsal medial magnocellular nuclei; MGN, medial geniculate nuclei; PuA, pulvinar anterior nuclei; PuI, pulvinar inferior nuclei; PuL, pulvinar lateral nuclei; PuM, pulvinar medial nuclei; VA, ventral anterior nuclei; VL, ventral lateral nuclei; VPL, ventral posterolateral nuclei.

According to the automated anatomical labeling atlas 3 (28), the dorsal thalamus of each hemisphere was subdivided into 15 subregions, which included the anteroventral, lateral posterior (LP), ventral anterior, ventral lateral (VL), ventral posterolateral, intralaminar (IL), mediodorsal medial magnocellular, mediodorsal lateral parvocellular, lateral geniculate, medial geniculate (MGN), pulvinar inferior, pulvinar medial, pulvinar anterior (PuA), and pulvinar lateral nuclei and reuniens. Each thalamic subregion was set as a region of interest (ROI) except for the reuniens, which were too small to be analyzed (Figure 1).

### Static and Dynamic FC Analyses

#### Static FC Analysis

For each participant, seed-based FC analysis was performed. Pearson's correlation coefficients between the mean time series of each thalamic ROI and the time series of every other voxel in the brain were computed and converted into *z*-values using Fisher's *r*-to-*z* transformation to improve normality.

#### Dynamic FC Analysis

Sliding time-window analysis was employed to characterize FC temporal dynamics. Previous studies have shown that a Hamming window length ranging from 30 s to 1 min (i.e., 15–30 TR) is suitable for capturing dynamic FC patterns (29, 30). The window length was set as 30 TR with a 1 TR step to obtain BOLD signal window time series of the preprocessed functional data of each participant. Seed-based FC analysis was performed for each window, and the means and standard deviations of the correlation maps across time windows were calculated for each subject (19, 31). Dynamic FC was determined by the standard deviation of the correlation maps. Higher dynamic FC variability was reflected by a larger standard deviation, which indicated greater fluctuations of FC strength.

### Statistical Analysis

The demographic and clinical data were analyzed using the Statistical Package for the Social Sciences version 19.0 (SPSS, Chicago, IL). The group difference in sex was compared using a chi-square test. Age, MMSE score, and FD were compared between the PD-FOG, PD-NFOG, and HC groups using a one-way analysis of variance (ANOVA). Illness duration, UPDRS III, and LEDD were compared between PD-FOG and PD-NFOG using a two-sample *t*-test. Hoehn-Yahr stage was compared between PD-FOG and PD-NFOG using a Mann-Whitney U test for ranked data. Static and dynamic FC analyses were conducted in a voxel-wise manner using SPM12. Group comparisons of static and dynamic FC between PD-FOG, PD-NFOG, and HC were performed for each thalamic ROI separately. Specifically, a one-way ANOVA was performed for the static and dynamic FC maps of a given thalamic seed to examine the differences in static or dynamic FC of each voxel within the whole brain to the seed. Corrections for multiple comparisons were performed using the cluster level family-wise error (FWE) method, which resulted in a cluster defining threshold of *p* < 0.001 and a corrected cluster significance of *p* < 0.05. If a cluster showed a significant inter-group difference, a *post hoc* two-sample *t*-test was performed to compare the mean FC value of this cluster between each group pair (*p* < 0.001). Pearson's correlation coefficients were calculated to examine the associations between the FC values extracted from significantly different brain regions and NFOGQ sores in PD-FOG patients. The significance level was set at *p* < 0.05 based on the exploratory nature of the correlation analyses.

### Validation Analysis

Because window length and cut-off filtered frequency were key parameters in the dynamic FC analysis, a window length of 100 s exceeding the longest BOLD signal wavelength was used, according to previous studies (32, 33). Based on the minimum filtered frequency of 0.01 Hz used in our study, we recalculated the dynamic FC maps for each thalamic seed using a window length of 50 TR. Ultimately, mean dynamic FC values with a window length of 50 TR were extracted from significant brain regions in the main analyses with window length as 30 TR and compared between PD-FOG, PD-NFOG, and HC in a ROI-wise manner.

## Results

### Demographic and Clinical Characteristics

Demographic and clinical data of the subjects are presented in Table 1. The three groups were well-matched for sex (chi-square test, χ^2^ = 0.427, *p* = 0.808), age (one-way ANOVA, *F* = 0.267, *p* = 0.766), MMSE score (one-way ANOVA, *F* = 2.705, *p* = 0.074), and FD (one-way ANOVA, *F* = 1.132, *p* = 0.328). There were no significant differences in durations of illness (two sample *t*-test, *t* = 0.740, *p* = 0.464), Hoehn-Yahr stage (Mann-Whitney U test, *Z* = − 0.643, *p* = 0.520), UPDRS III (two sample *t*-test, *t* = 0.096, *p* = 0.924), and LEDD (two sample *t*-test, *t* = 1.978, *p* = 0.054) between PD-FOG and PD-NFOG groups.

**Table 1:** Demographic and clinical characteristics.

**Characteristics**	**PD-FOG (*n* = 25)**	**PD-NFOG (*n* = 25)**	**HC (*n* = 25)**	**Statistics**	***P-*value**
Sex (male/female)	13/12	13/12	11/14	*χ^2^* = 0.427	0.808^a^
Age (years)	65.320 ± 8.385	65.240 ± 6.796	63.880 ± 8.212	*F* = 0.267	0.766^b^
MMSE	26.000 ± 2.517	26.120 ± 1.878	27.200 ± 1.500	*F* = 2.705	0.074^b^
FD (mm)	0.104 ± 0.084	0.082 ± 0.044	0.081 ± 0.035	*F* = 1.132	0.328^b^
Illness duration (years)	7.440 ± 4.435	6.700 ± 2.309	NA	*t* = 0.740	0.464^c^
Hoehn-Yahr stage	2.600 ± 0.791	2.460 ± 0.721	NA	*Z* = −0.643	0.520^d^
UPDRS III	33.320 ± 15.247	32.960 ± 10.964	NA	*t* = 0.096	0.924^c^
LEDD (mg)	466.000 ± 179.971	377.500 ± 132.925	NA	*t* = 1.978	0.054^c^
NFOGQ	20.520 ± 5.539	NA	NA	NA	NA

*Data are shown as means ± standard deviations. FD, frame-wise displacement; HC, healthy controls; LEDD, levodopa equivalent daily dose; MMSE, mini-mental state examination; NA, not applicable; NFOGQ, new freezing of gait questionnaire; PD-FOG, Parkinson's disease patients with freezing of gait; PD-NFOG, Parkinson's disease patients without freezing of gait; UPDRS III, Unified Parkinson's Disease Rating Scale part III*.

a *Chi-square test was used to test differences in sex between the three groups*.

b *One-way ANOVA was used to test differences in age, MMSE score, and FD between the three groups*.

c *Two-sample t-tests were used to compare illness duration, UPDRS III score, and LEDD between the two PD patient groups*.

d *Mann-Whitney U test was used to compare the Hoehn-Yahr stage between the two PD patient groups*.

### Static FC

**Figure 2:**
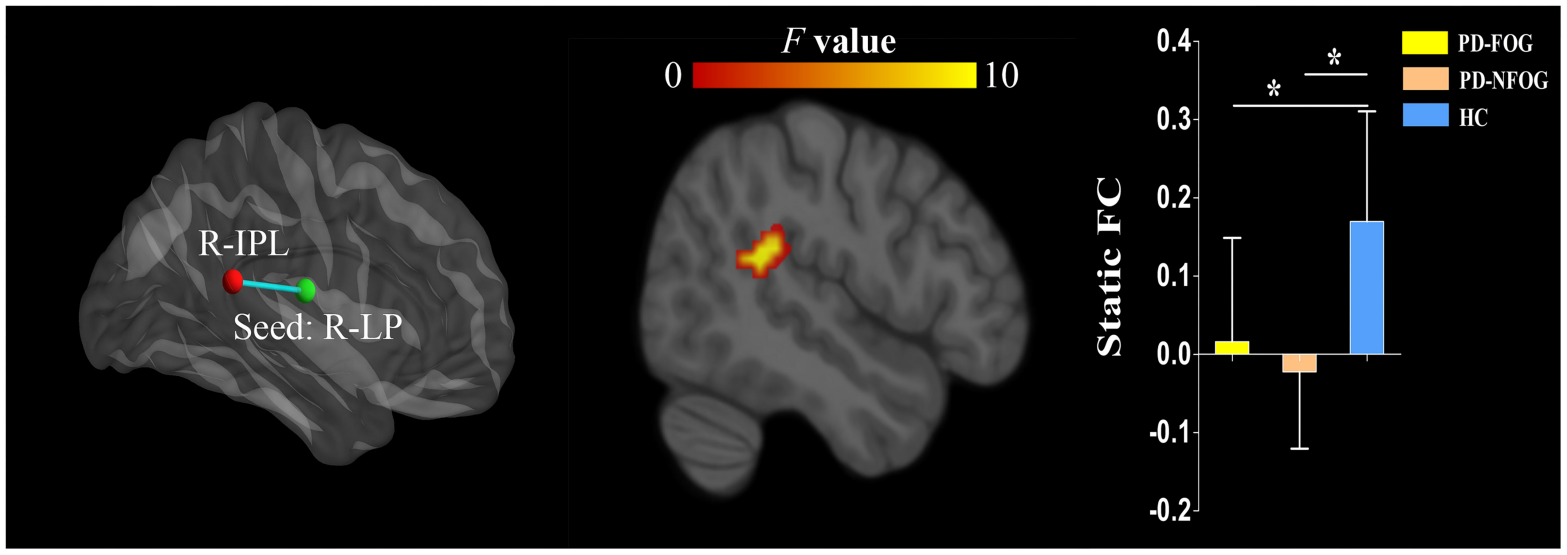
Inter-group differences in static FC of the thalamic nuclei between PD-FOG, PD-NFOG, and HC. FC, functional connectivity; HC, healthy controls; IPL, inferior parietal lobule; LP, lateral posterior nuclei; PD-FOG, Parkinson's disease patients with freezing of gait; PD-NFOG, Parkinson's disease patients without freezing of gait; R, right; *p < 0.001.

The ANOVA showed significant static FC differences in the right LP nuclei and right inferior parietal lobule (IPL, cluster size = 72 voxels, peak MNI coordinate x/y/z = 48/−42/18, peak *F* = 13.302) between the three groups (*p* < 0.05, cluster-level FWE corrected). *Post-hoc* analyses revealed that compared with HC, PD-FOG and PD-NFOG exhibited lower static FC between the right LP nuclei and right IPL (Figure 2). No other thalamic nuclei showed group differences in static FC.

### Dynamic FC

**Figure 3:**
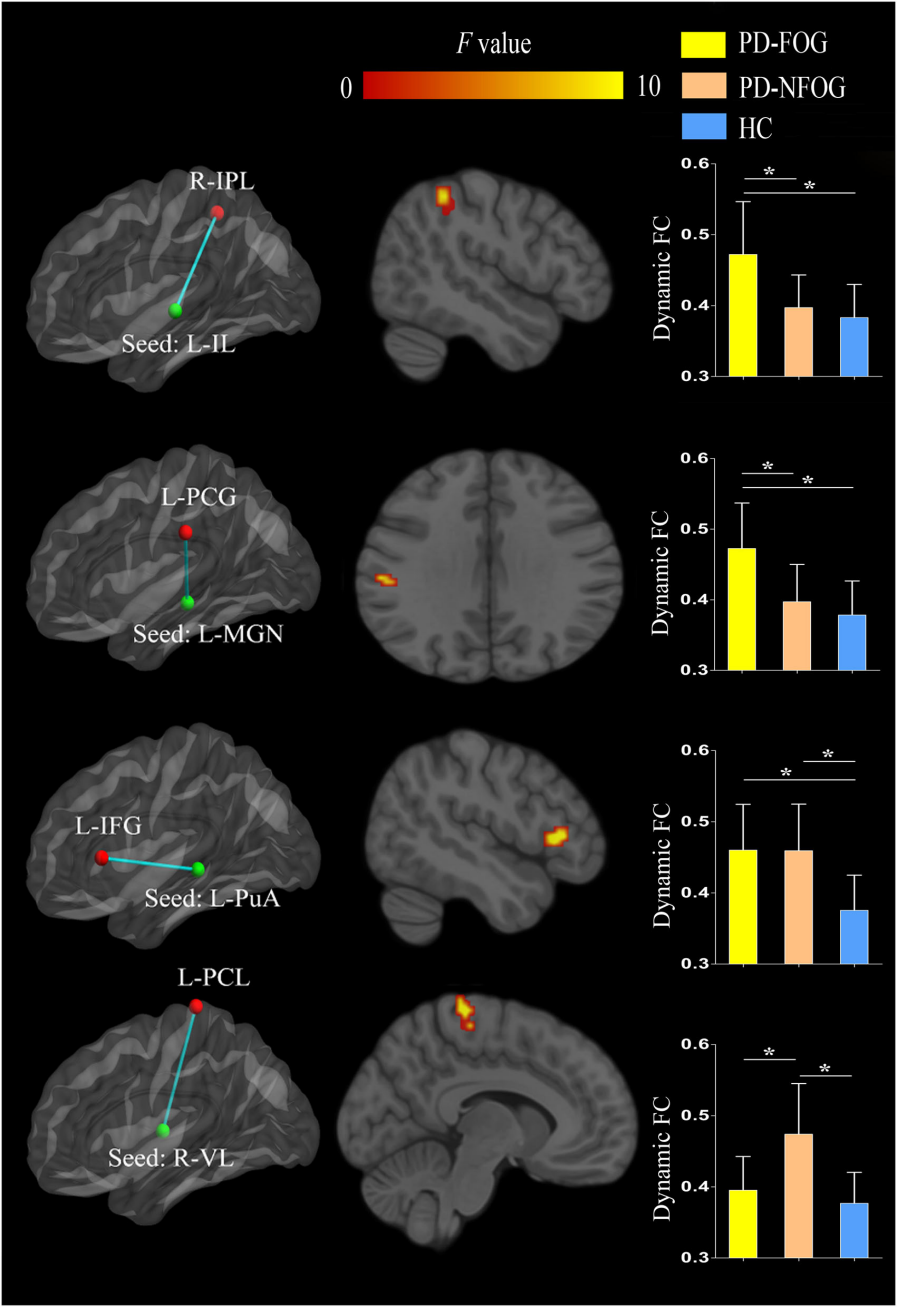
Inter-group differences in dynamic FC of the thalamic nuclei between PD-FOG, PD-NFOG, and HC. FC, functional connectivity; HC, healthy controls; IFG, inferior frontal gyrus; IL, intralaminar nuclei; IPL, inferior parietal lobule; L, left; MGN, medial geniculate nuclei; PCG, postcentral gyrus; PCL, paracentral lobule; PuA, pulvinar anterior nuclei; PD-FOG, Parkinson's disease patients with freezing of gait; PD-NFOG, Parkinson's disease patients without freezing of gait; R, right; VL, ventral lateral nuclei; *p < 0.001.

The ANOVA revealed significant inter-group differences in dynamic FC between the left IL nuclei and right IPL (cluster size = 21 voxels, peak MNI coordinate x/y/z = 48/−42/51, peak *F* = 11.290), between the left MGN and left postcentral gyrus (PCG, cluster size = 15 voxels, peak MNI coordinate x/y/z = −57/−24/30, peak *F* = 11.277), between the left PuA nuclei and left inferior frontal gyrus (IFG, cluster size = 18 voxels, peak MNI coordinate x/y/z = −48/24/6, peak *F* = 15.315), and between the right VL nuclei and left paracentral lobule (PCL, cluster size = 22 voxels, peak MNI coordinate x/y/z = −9/−30/72, peak *F* = 16.583; *p* < 0.05, cluster-level FWE corrected). *Post-hoc* analyses revealed that PD-FOG showed higher dynamic FC between the left IL nuclei and right IPL and between the left MGN and left PCG than that of the PD-NFOG and HC. PD-FOG and PD-NFOG patients exhibited higher dynamic FC between the left PuA nuclei and left IFG. However, PD-NFOG patients showed higher dynamic FC between the right VL nuclei and left PCL relative to that of the PD-FOG and HC (Figure 3).

### Associations Between FC and Clinical Characteristics in the PD-FOG Group

**Figure 4:**
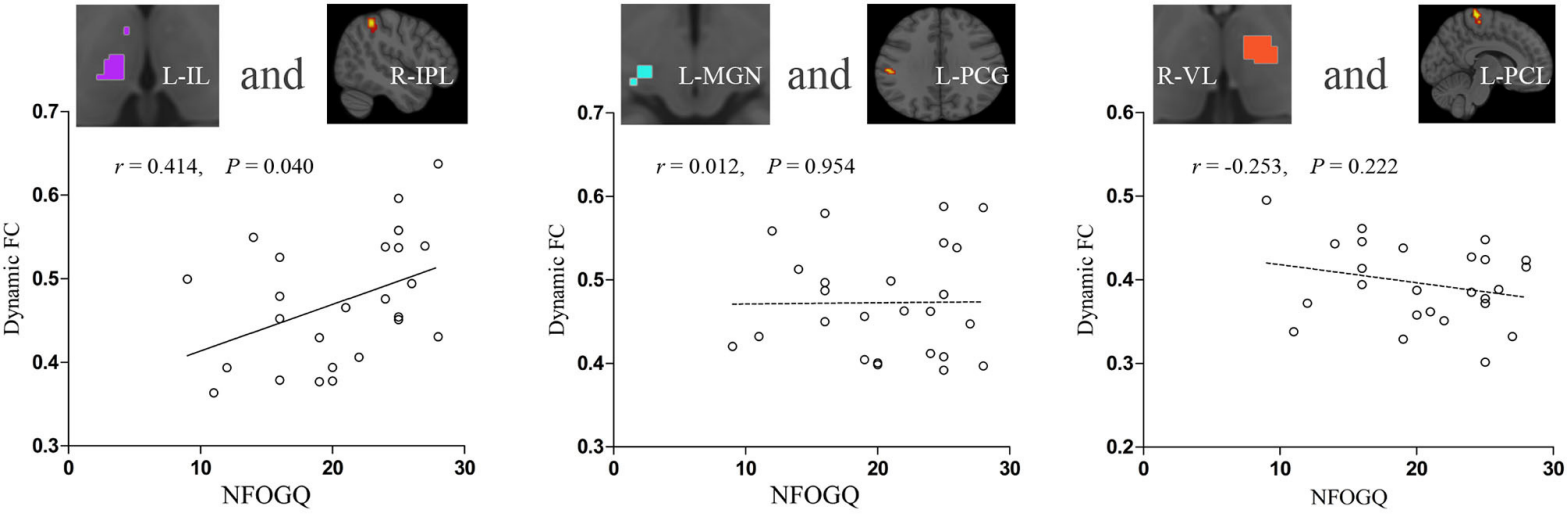
Correlations between increased thalamic dynamic FC and symptom severity in PD-FOG. FC, functional connectivity; IL, intralaminar nuclei; IPL, inferior parietal lobule; L, left; MGN, medial geniculate nuclei; NFOGQ, new freezing of gait questionnaire; PCG, postcentral gyrus; PCL, paracentral lobule; PD-FOG, Parkinson's disease patients with freezing of gait; R, right; VL, ventral lateral nuclei.

In PD-FOG, dynamic FC between the left IL nuclei and right IPL was positively correlated with the NFOGQ score (*r* = 0.414, *p* = 0.040). No correlation of dynamic FC with NFOGQ score was identified in other thalamic nuclei with inter-group dynamic FC differences between PD-FOG and PD-NFOG (Figure 4).

### Validation Analysis

We found that the inter-group differences of dynamic FC, which were identified using a window length of 30 TR, were still present when using a window length of 50 TR (Supplementary Figure 1).

## Discussion

The present study examined static and dynamic FC between the thalamic nuclei and other brain areas using seed-based analyses of resting-state fMRI in PD-FOG and PD-NFOG. The results revealed that dynamic FC between multiple thalamic nuclei and cerebral cortex is disrupted in PD patients, with distinct profiles between PD-FOG and PD-NFOG. The thalamic nuclei identified with altered dynamic FC included associative nuclei (IL, PuA) and relay nuclei (MGN, VL) which are functionally associated with cognition and sensorimotor processes. The altered dynamic FC between the thalamic nuclei and cerebral cortex that was specific to PD-FOG was between the left IL nuclei and right IPL and between the left MGN and left PCG. Moreover, dynamic FC between the left IL nuclei and right IPL correlated with the severity of FOG. To the best of our knowledge, this is the first resting-state fMRI study investigating static and dynamic FC of the thalamus using individual thalamic nuclei as ROI and exploring the association of dynamic FC alterations with FOG symptoms in PD patients.

### Static FC Between the Thalamic Nuclei and Other Brain Regions in PD

Static FC reflects the average connectivity strength between regions over time. Our results showed lower static FC between the right LP nuclei and right IPL in PD patients compared with HC, regardless of the presence of FOG, indicating this alerted FC is related to Parkinsonian features other than FOG. The LP nuclei belong to the associative group of thalamic nuclei. They have reciprocal connections with the parietal lobe and receive inputs from the superior colliculus, cingulate gyrus, and parahippocampal cortex. The exact functions of the LP nuclei are not well understood but are thought to function together with the pulvinar nuclei of the thalamus for the integration of sensorimotor and spatial attention. The IPL is a multimodal brain region that is subdivided into several cytoarchitectonic areas and connected to the somatomotor areas, lateral frontal cortex, and regions of the default mode network. It is involved in the neural networks related to spatial attention, sensorimotor processing, movement imagination, audition perception, and inhibitory control. The disturbed static FC between right LP nuclei and right IPL seemed to suggest an impairment of sensorimotor integration or spatial attention in PD patients.

Previous FC investigations in PD implicating the thalamus have yielded inconsistent results with research demonstrating increased coupling between the thalamus and sensorimotor regions (34) or no significant alteration in thalamic FC (35). More recently, a seed-based fMRI FC study demonstrated that PD is associated with increased static FC between the motor subdivisions of the thalamus (VL/ventral anterior nuclei) and the supplementary motor area and between the prefrontal thalamic subdivisions (mediodorsal and anterior nuclei) and the basal ganglia nuclei or anterior and dorsolateral prefrontal cortices (36). A seed-to-voxel static FC study performed with the seeds in the bilateral basal ganglia nuclei, thalamus, and pedunculopontine nucleus revealed that FC of bilateral thalamus and globus pallidus external with visual cortex was significantly increased in PD-FOG compared to PD-NFOG (15). The discrepancy between our study results and previous reports may be attributed to differences in the placement of ROIs and subjects recruited.

### Dynamic FC Between the Thalamic Nuclei and Other Brain Regions in PD

Dynamic FC reflects the fluctuation of brain connectivity strength over time and detects fluctuations in FC from the spontaneous brain activity of various neural substrates more comprehensively. However, dynamic FC changes in the context of neural functioning remain poorly understood; higher dynamic FC between brain regions may be indicative of flexible communication, but it may also be a sign of unstable interactions. Static and dynamic FC methods capture different aspects of inter-regional communication and provide complementary information. In the current study, altered dynamic FC was observed in more thalamic nuclei than observed with static FC in the same cohort of PD patients, indicating that dynamic FC may be more sensitive than static FC in detecting thalamic functional abnormality.

Our study identified two thalamic nuclei with increased fluctuation of dynamic FC with specific cortex areas in PD-FOG but not in PD-NFOG. The first was between the left IL nuclei and the right IPL. The IL nuclei are composed of two sub-groups within the internal medullary lamina: the anterior (rostral) and posterior (caudal) nuclei (37). The anterior group consists of the central medial, paracentral, and central lateral nuclei, having reciprocal connections with widespread associative cortical areas and also receiving projections from subcortical structures including brainstem reticular formation, spinothalamic pathways, and superior colliculus. The posterior group, composed of centromedian and parafascicular nuclei, have reciprocal connections with the motor cortex and connected with several important subcortical motor regions such as internal globs pallidus/pars reticulata of substantia nigra, pedunculopontine nucleus, and subthalamus and deep cerebellar nuclei. Both the anterior and posterior groups of the IL nuclei project efferent fibers to the striatum. The complex anatomic connections indicate that IL nuclei have a wide spectrum of modulating functions including wakefulness maintaining, sensory-motor integration, and numerous cognitive functions (38). The mechanism by which the IL nuclei modulate cortical functions is poorly understood. The IL nuclei are part of the higher-order thalamus, which receive little direct sensory inputs, and instead have reciprocal connections with widespread cortical areas and form extensive thalamocortical pathways. It was hypothesized that IL nuclei and other higher order thalamic nuclei modulated cortical information transmission and cognitive processing by adjusting cortical synchrony and oscillatory patterns and thereby the efficacy of information transmission (39). As mentioned above, the IPL is a brain region connected to a variety of networks, including sensorimotor network, the default mode network, the frontoparietal control network, and the cingulo-opercular network, and functionally involved in spatial attention, sensorimotor processing, self-perception, and social cognition. Given the structural and functional links between the IL nuclei and IPL (40, 41), the increased dynamic FC between these two areas in PD-FOG suggests altered interactions between the thalamus and cortex which is related to sensorimotor integration and/or cognitive modulation of motor function. Indeed, FOG appears to be closely related to attentional and executive dysfunction as well as impaired sensorimotor integration. The greater temporal fluctuation of this crucial circuitry suggests that PD subjects rely more on sensory feedback and executive cognitive control during motor performance. The cross-hemispheric dynamic FC alteration between the left IL nuclei and the right IPL is an interesting phenomenon. It is well known that interhemispheric coordination through the corpus callosum is crucial for some motor functions, especially for gait, since locomotion movement involves bilateral limbs. Reduced interhemispheric connectivity has been proposed to play a role for FOG in PD (16). The ventral attention network, comprising the right IPL and the ventral frontal cortex and being responsible for detecting unexpected stimuli and inducing attentional shifts in cooperation with the dorsal system, is deemed more right lateralized (42, 43). As discussed above, attention and execution dysfunctions are closely related to the development of FOG in PD. Taken together, the increased dynamic FC between the left IL nuclei and the right IPL suggested that the left IL nuclei in cooperation with the right lateralized ventral attention network through interhemispheric connection may play a role in the pathophysiology of FOG. The disrupted dynamic FC between the IL nuclei and IPL may not result exclusively from the degeneration of midbrain dopaminergic neurons and subsequent interruption of the gait control network. A rich deposition of alpha-synuclein aggregates in Lewy neurites accumulates in the IL regions in PD patients at postmortem but spares most other regions of the thalamus (44). The pathological lesion in IL may contribute to the interrupted dynamic FC of IL nuclei with IPL.

The second thalamic nucleus that showed greater dynamic FC fluctuations with the left PCG in PD-FOG but not PD-NFOG was the MGN. The MGN belongs to the sensory relay nuclei of the thalamus and receives ascending auditory fibers that project to the primary auditory cortex in the temporal lobe. The PCG is the primary somatosensory cortex, and extensive ipsilateral connections and interactions between the primary auditory cortex and primary or secondary somatosensory regions have been demonstrated in humans by neuroimaging study (45). The links between the auditory and somatosensory cortices provide anatomical and functional bases for the alteration of dynamic FC between the MGN and PCG observed in PD-FOG in the present study. This finding is consistent with previous studies about the sensory cuing effects on FOG. Inputs from a variety of sensory systems have been shown to either exacerbate or alleviate FOG (46), and the use of cueing therapy can temporarily improve freezing behavior (47). Rhythmic auditory stimulation increases the consistency of gait, increases automaticity, and decreases gait variability (48). Moreover, external auditory cues can help to maintain gait performance and decrease interference during tests that require dual-tasking (49). Furthermore, lesions to multiple brain locations, including the PCG and other frontal areas, have been reported to cause FOG (50), which is consistent with our finding of altered dynamic FC of the PCG in PD-FOG. Increased dynamic FC between the MGN and PCG in PD-FOG may be a reflection of compensatory adaptation, although it may also be a consequence of impaired sensorimotor integration.

Increased dynamic FC fluctuations between the left PuA nuclei and IFG were detected in both PD-FOG and PD-NFOG, indicating that it is associated with general neural dysfunction in PD, rather than a FOG related specific alteration. The pulvinar are major multimodal associational nuclei in the thalamus, receiving inputs from the visual pathway and projecting to the visual cortex, as well as to association areas of the parietal, temporal, and prefrontal cortices. The exact function of the pulvinar is unclear. However, based on its connections, it is suggested that it is involved in multisensory integration and visually modulated higher functions (perception, attention orienting, and selection). A fMRI study exploring thalamus-related cortical networks demonstrated that the pulvinar nuclei are involved in both the dorsal attention and visual networks (51). The pulvinar nuclei can be divided into four subdivisions (anterior, medial, lateral, and inferior) with distinct connections and functions. The PuA subdivision is connected to the somatosensory cortex and is functionally involved in sensorimotor functions, especially motor selection and programming functions (52). Moreover, PuA nuclei are associated with networks implicated in cognition, such as language and task-related performance (53, 54), which suggests that the increased dynamic FC fluctuations between the left PuA nuclei and IFG observed in the PD patients in our study may be associated with motor planning and programming dysfunctions.

Increased dynamic FC fluctuations between the right VL nuclei and left PCL were observed in PD-NFOG but not in PD-FOG. The VL nuclei are well-established motor relays in the thalamus, receiving input from the basal ganglia and cerebellum and projecting to the motor cortex. The ventral lateral nucleus is the shared node of two important motor circuits, namely the cortico-basal ganglia-thalamocortical circuit and cortico-cerebello-thalamocortical circuit and is active during both passive and active movements of the contralateral half-body. Increased static FC between the VL/ventral anterior nuclei of the thalamus and the supplementary motor area has been observed in PD patients in a seed-based fMRI study (36). We did not replicate this static FC alteration but identified increased fluctuation of dynamic FC between VL nuclei of the thalamus and PCL in PD-NFOG. However, this altered dynamic FC was not detected in PD-FOG, indicating there existed compensatory adaption that normalized the increased dynamic FC between VL nuclei and PCL in the FOG subgroup of PD patients.

### Associations Between FC and the Severity of FOG in PD-FOG

Among the altered dynamic FC between the thalamic nuclei and cortical areas, only the dynamic FC between the left IL nuclei and right IPL was identified to be positively correlated with the severity of FOG, while other thalamic nuclei-cortex dynamic FC with intergroup differences between PD-FOG and PD-NFOG were not correlated with NFOGQ sores. The result suggested that the disrupted dynamic FC between the IL nuclei and IPL was involved in the physiopathologic mechanism of FOG and may serve as a potential neuroimaging marker.

There are several limitations to this study. First, the pathophysiological mechanisms or causal relationship of the altered FC between the thalamic nuclei and certain cortical regions identified in the current study in the development of FOG in PD cannot be determined. Actually, this is the major limitation faced by most neuroimaging studies. Nevertheless, our findings provided meaningful clues for further study with respect to the FC changes in the physiopathology underlying FOG. Second, the sample size was modest and potential medication effect may increase the instability of results. Future studies in a larger sample of drug-naive patients with PD are needed to confirm our findings, which would further enhance our understanding of the role of the thalamus in FOG. Third, the severity of FOG was assessed using questionnaires which may not be sufficiently sensitive and accurate in measuring FOG severity. This may have limited the discovery of significant correlations between the FC measures and clinical variables. A more objective evaluation method of FOG such as the freezing index detected by gait analysis device may help to overcome the problem. Lastly, altered dynamic FC between the left IL nuclei and right IPL and between the left MGN and left PCG have been speculated to be associated with attention/execution and sensorimotor integration in our study. However, its correlations with relevant clinical parameters have not been examined. Further studies should include comprehensive tests with regard to attention/execution function and auditory cuing effects on FOG.

In conclusion, our findings revealed that dynamic FC between multiple thalamic nuclei and the cerebral cortex are altered in PD patients. PD-FOG had a specific profile of dynamic FC disruption compared with that of PD-NFOG, suggesting that altered dynamic FC between the left IL nuclei and right IPL and between the left MGN and left PCG is associated with the development of FOG in PD patients. Our study provides further evidence for the pivotal role of the thalamus in the neuropathology of FOG in PD patients.

## Data Availability Statement

The original contributions presented in the study are included in the article/Supplementary Material, further inquiries can be directed to the corresponding authors.

## Ethics Statement

The studies involving human participants were reviewed and approved by The First Affiliated Hospital of Anhui Medical University. The patients/participants provided their written informed consent to participate in this study.

## Author Contributions

All co-authors meet the criteria for authorship. SW, HC, XC, and YY designed the study and wrote the draft of the manuscript. SW, HC, and ZC performed the image processing and statistical analyses. CL, TW, and XC undertook neurological assessments. YQ and ZC operated the MRI machine. FX conducted the literature search. All authors contributed to and have approved the final manuscript.

## Funding

This work was supported by grants from the National Natural Science Foundation of China (Grant nos: 81971072, 81771817, and 82071905).

## Conflict of Interest

The authors declare that the research was conducted in the absence of any commercial or financial relationships that could be construed as a potential conflict of interest.

## Publisher's Note

All claims expressed in this article are solely those of the authors and do not necessarily represent those of their affiliated organizations, or those of the publisher, the editors and the reviewers. Any product that may be evaluated in this article, or claim that may be made by its manufacturer, is not guaranteed or endorsed by the publisher.
