# Supplementary material for: More Than Just Static: Dynamic Functional Connectivity Changes of the Thalamic Nuclei to Cortex in Parkinson's Disease With Freezing of Gait
Source: Front Neurol. 2021 Oct 15;12:735999. doi: 10.3389/fneur.2021.735999 (PMC8553931; doi:10.3389/fneur.2021.735999)
Supplement: Supplementary file 1 [file Image_1.pdf]

Supplementary Material

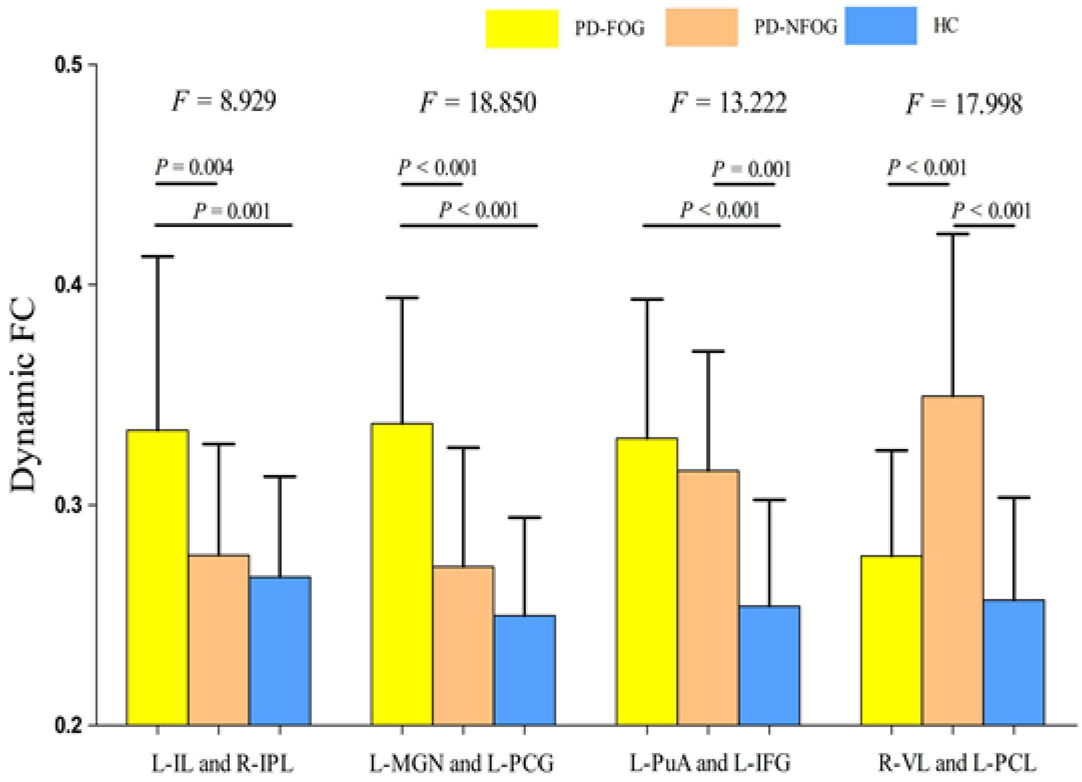

Supplementary Figure 1. Dynamic functional connectivity changes among PD-FOG, PD-NFOG and HC identified using a window size = 50 TR. Abbreviations: FC, functional connectivity; HC, healthy controls; IFG, inferior frontal gyrus; IL, intralaminar nuclei; IPL, inferior parietal lobule; L, left; MGN, medial geniculate nuclei; PCG, postcentral gyrus; PCL, paracentral lobule; PD-FOG, Parkinson's disease patients with freezing of gait; PD-NFOG, Parkinson's disease patients without freezing of gait; PuA, pulvinar anterior nuclei; R, right; VL, ventral lateral nuclei.
